# Closing the Performance Gap between Siamese Networks for Dissimilarity Image Classification and Convolutional Neural Networks

**DOI:** 10.3390/s21175809

**Published:** 2021-08-29

**Authors:** Loris Nanni, Giovanni Minchio, Sheryl Brahnam, Davide Sarraggiotto, Alessandra Lumini

**Affiliations:** 1 Department of Information Engineering (DEI), University of Padova, 35131 Padova, Italy; gianluca.maguolo@phd.unipd.it (G.M.); davide.sarraggiotto@studenti.unipd.it (D.S.); 2 Department of Information Technology and Cybersecurity, Missouri State University, 901 S, National Street, Springfield, MO 65804, USA; sbrahnam@missouristate.edu; 3 Department of Computer Science and Engineering (DISI), University of Bologna, Via dell’Università 50, 47521 Cesena, Italy; alessandra.lumini@unibo.it

**Keywords:** Siamese networks, ensemble of classifiers, loss function, discrete cosine transform

## Abstract

In this paper, we examine two strategies for boosting the performance of ensembles of Siamese networks (SNNs) for image classification using two loss functions (Triplet and Binary Cross Entropy) and two methods for building the dissimilarity spaces (FULLY and DEEPER). With FULLY, the distance between a pattern and a prototype is calculated by comparing two images using the fully connected layer of the Siamese network. With DEEPER, each pattern is described using a deeper layer combined with dimensionality reduction. The basic design of the SNNs takes advantage of supervised k-means clustering for building the dissimilarity spaces that train a set of support vector machines, which are then combined by sum rule for a final decision. The robustness and versatility of this approach are demonstrated on several cross-domain image data sets, including a portrait data set, two bioimage and two animal vocalization data sets. Results show that the strategies employed in this work to increase the performance of dissimilarity image classification using SNN are closing the gap with standalone CNNs. Moreover, when our best system is combined with an ensemble of CNNs, the resulting performance is superior to an ensemble of CNNs, demonstrating that our new strategy is extracting additional information.

## 1. Introduction

Interest in classification systems based on (dis)similarity spaces is resurging. Unlike the more common technique of classifying samples within a feature space, (dis)similarity classification estimates the class of an unknown pattern by examining its similarities and dissimilarities with a set of training samples and pairwise (dis)similarities between each of the members. This process has come to involve more than the application of standard distance measures; (dis)similarity classification is also a way to build new spaces.

Though the two terms of similarity and dissimilarity are rarely disambiguated in the literature, classification based on the notion of dissimilarity is an idea first proposed in [1], where the focus was on comparing differences between samples belonging to different classes. Dissimilarity classification can be tackled by using either dissimilarity vectors, as in [2,3,4,5,6], or dissimilarity spaces, as in [7,8,9,10,11,12,13,14]. In the former case, two samples are considered positive if they belong to the same class and negative if they belong to separate classes. The goal of the classifier is to decide which of these two cases a given vector was calculated on. For a more detailed discussion of this approach, see [15].

In contrast, dissimilarity methods that generate dissimilarity spaces, the approach taken here, produce classifiers from within feature vector spaces. Unlike traditional feature vectors representing samples as measured across all features, representation from feature vector spaces is the distance between pairs of samples. In [1], which introduced this approach, the authors applied prototype selection for training classifiers on dissimilarity spaces. The dissimilarity representations were used as a vector space. This method was applied to image retrieval by [8] using a prototype-based dissimilarity space. In [10], a compact representation based on prototype selection methods was derived from deep convolutional features and learned distance measures.

A loss function commonly used in dissimilarity classification is the Maximum Mean Discrepancy (MMD). In [11], the application of MMD enabled the source and target data in the dissimilarity space to harness the intra-class and inter-class distributions to produce a pairwise matcher. This version of MMD was also shown to work well across several data sets. A modification of the contrastive loss function for a Siamese Neural Network (SNN) [16,17] was proposed in [18] for brain image classification. The correlation distance of this variant of the loss function predicted the output features of image pairs. This method was expanded for audio classification in [12,13]. The audio samples, represented as spectrograms, were transformed by clustering methods into a set of centroids that generated dissimilarity spaces via SNN. The audio samples were then projected into the dissimilarity spaces to obtain a vector space representation that could be used to train Support Vector Machines (SVMs). An improved version of this method was developed for generic image classification in [14], where dissimilarity spaces were produced by a set of clustering methods and a set of SNNs with different CNN backbones. This approach was shown to compete well against state-of-the-art classifiers on several image data sets and obtained the highest classification score on one of them.

This work further expands [14] by proposing additional techniques for improving the performance of an ensemble of SNNs. As in the earlier work, each Siamese network, composed of eight different CNN topologies, generates a dissimilarity space whose features train an SVM, and the SVMs are then combined by sum rule. The strategies investigated here for improving performance further are the following:

- Two different loss functions are used to train the Siamese networks: the binary cross entropy loss function and the triplet loss function.
- Two different approaches for building the dissimilarity spaces are proposed for extracting features: the first is based on the fully connected layer and the latter on a deeper layer where the size of each channel is reduced by the Discrete Cosine Transform (DCT).
- SNNs are optimized using different variants of Adam, with a new Adam variant proposed in this work.

Systems built with these strategies are compared, fused, and evaluated with previous work on dissimilarity classification. The versatility and robustness of the best ensemble developed using these techniques are demonstrated on five cross-domain image data sets representing medical imaging problems, animal vocalizations (spectrograms), and portrait images.

## 2. Proposed Approach

The basic system can be described as follows. The inputs into the system, as in [12,13,14], are the original images and HASC descriptors [19], extracted to produce a new processed image. If the original image is in color, Hasc is applied separately on each band; if it is grey level, the Hasc image is replicated three times to build an image with three bands.

**Figure 1:**
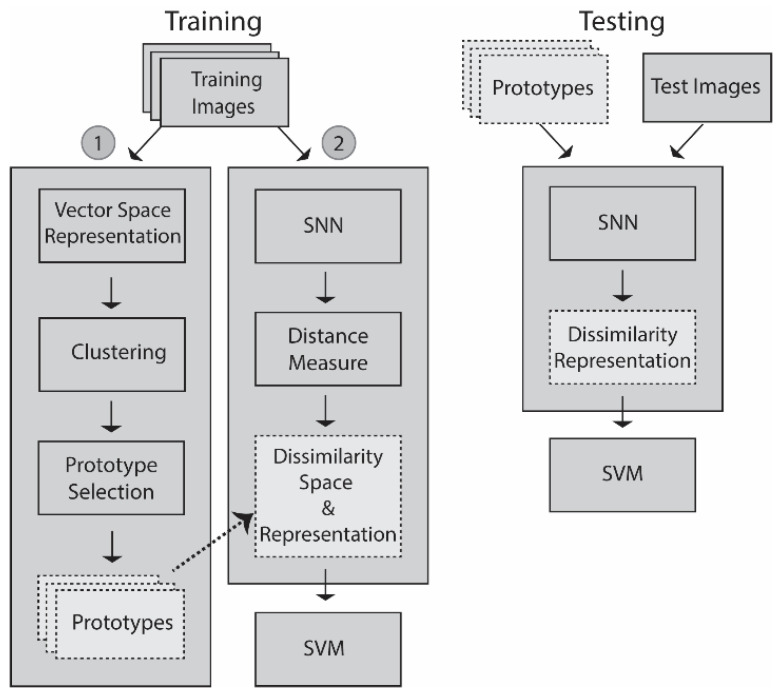
Schematic of the basic dissimilarity architecture using one SNN with the output fed into one SVM.

Starting with the vector space representations, step 1 of the training process, as illustrated in Figure 1, begins by generating a set of clusters that produce a set of prototypes. The prototypes are centroids generated by k-means on the vector space representations. In step 2, a dissimilarity space is generated by an SNN that learns a distance measure from the prototypes that maximizes differences between pairs within class while also minimizing differences of pairs between other classes, a process that produces a feature vector that is trained on an SVM. In the testing stage, an unknown pattern is projected onto the dissimilarity space that was learned by the SNN, which generates the feature vector that is then fed into the trained SVM (we have not optimized the SVM hyperparametes, we have used a generic setting: Radial basis function kernel; C = 1000; gamma = 0.1) for a decision.

**Figure 2:**
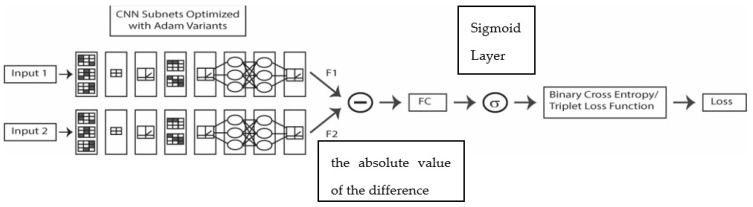
Schematic of SNN.

The SNN, as illustrated in Figure 2, combines two identical deep learners whose outputs are subtracted, which produces a feature vector (the absolute value of the difference) that is passed to a sigmoid and a loss function as in [12,13,14]. In this way, the FC layer and sigmoid predict the dissimilarity of the two input images (Inputs 1 and 2). The feature vector (*FC*) is computed by subtracting the outputs (*F*1 and *F*2) as follows:

FC=|F1−F2|

Unlike [12,13,14], which used binary cross entropy, two different loss functions are tested here (binary cross entropy and triplet loss function), and the CNN subnets are optimized with Adam and some Adam variants.

Though some variations are indicated in Figure 1 and Figure 2, they only show the output of one SNN fed into one SVM. In [12,13,14] and this work, many SNNs and SVMs are trained, tested, and combined. Eight CNN topologies form the backbone of the SNNs. These are the identical topologies described in [14] (for the reader’s convenience, the table in [14] that details the topologies is reprinted in the Appendix A). Thus, a large number of SNNs are trained using the different topologies, the two loss functions, and the Adam optimization algorithms. Each of these systems is tested, fused, and evaluated to build the best-performing system empirically.

The pseudocode for each step in Figure 1 can be found in the following sources: [12,13,14] (see as well the companion source code for this paper available at https://github.com/LorisNanni (accessed on 25 August 2021)).

Below, we focus on the new techniques proposed in this work: the application of two methods for generating the dissimilarity space (Section 2.1), the two different loss functions (Section 2.2) and the Adam optimization methods, including a new one proposed here (Section 2.3).

### 2.1. Methods for Generating the Dissimilarity Spaces

Both methods for generating the dissimilarity space follow the same basic process used in [12,13,14]: first, k-means is applied on a vector space representation of the training images, with prototypes calculated as the k centroids of the clusters produced. Second, a feature vector $F\;\in \;R_{k}$ is extracted by calculating the distances of image x from each of the prototypes, where the distance for each $F_{i}$ between x and prototype $p_{i}$ is given as $F_{i}=d\left(x,\;p_{i}\right).$ The resulting feature vector $F_{i}$ is fed into the SVM.

The two methods for generating the dissimilarity space are labeled FULLY and DEEPER. With FULLY, the distance between a pattern and a prototype is obtained directly by comparing the two images using the Siamese network. With DEEPER, each pattern is described using a deeper layer than the fully connected backbone network of the Siamese network. To reduce the high dimensionality of this deeper layer, the Discrete Cosine Transform (DCT) is applied separately to each channel of that layer (see Section 2.2). Finally, the distance between a pattern and a prototype is given by the cosine distance. In other words, the backbone of the Siamese network is used as the feature extractor.

**Figure 3:**
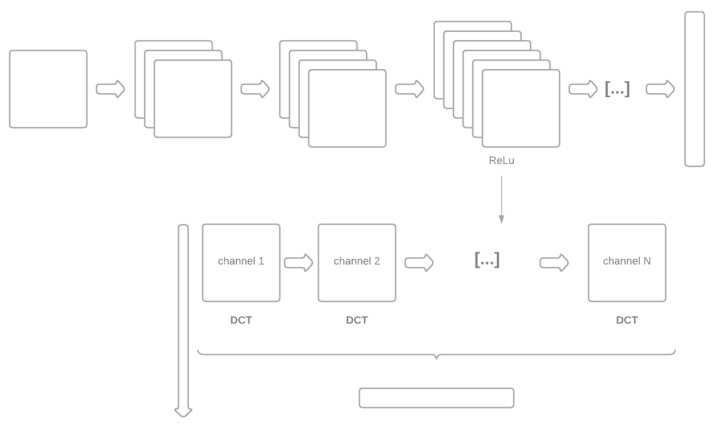
Schematic of DEEPER.

For the sake of space, the layers used in DEEPER are reported in the MATLAB toolbox available at https://github.com/LorisNanni (accessed on 25 August 2021) (for the reader’s convenience, these layers are also reported in the Appendix A of this paper). This step is not optimized. We have chosen the layer before the last ReLu or fully connected layer to prevent overfitting the results rather than selecting layers optimized for each data set. Optimal layers could have been discovered using a leave-one-out data set, but this procedure was not feasible given the computational power of our GPUs. In Figure 3 we report the scheme of DEEPER.

#### DCT Dimensionality Reduction

Because DEEPER uses a deeper layer compared to the fully connected backbone to generate the dissimilarity space, a method is needed to reduce dimensionality on each channel (with results combined) of the deeper layer. DCT [20] is the dimensionality transform selected here because (1) its components are typically small in magnitude (most information is located in the low-frequency coefficients), and (2) it balances information packing and computational complexity.

*DCT* can be expressed as

(1) $$DCTimage\left(x,y\right)=\frac{1}{\sqrt{2N}}C\left(x\right)C\left(y\right)\sum_{p,q=1}^{N}Image\left(p,q\right)\cos\frac{\left(2p+1\right)x\pi }{2N}\cos\frac{\left(2q+1\right)y\pi }{2N},$$

$$C\left(u\right)=\{\begin{array}{cc} \sqrt{\frac{1}{2}}, & u=0\\ 1, & u>0 \end{array}$$

where N is the number of row/columns of the image (input of CNN is a square matrix); p and q are the pixel indices of the input image; x and y are the indices of the *DCT* matrix.

Each channel is reduced to a dimension of 9 × 9. All the features extracted from each channel are concatenated into a single vector that represents a given pattern/prototype.

### 2.2. Loss Functions

#### 2.2.1. Binary Cross Entropy Loss (Cross)

In the training phase, every pair of images in the training set is fed into the backbone of the Siamese architecture to obtain a feature vector F. Calculated next is $Z=\left|F_{1}-F_{2}\right|$, where $F_{1}$ and $F_{2}$ are the feature vectors of the two images in the pair. Z is passed through a fully connected layer and a sigmoid function that returns the probability Y that the two images belong to the same class. Cross is then used for the two-class problem.

In the testing phase, for every sample in the training set, we compute F. Then, we evaluate N centroids using k-means clustering. Every image in the training set is expressed as the vector of the distances between its features and the centroids. After that, we train an SVM on those vectors. We then apply this inference algorithm to the images in the test set.

#### 2.2.2. Triplet Loss (Triplet)

With Triplet, we take three images as the inputs, labelled *A*, *P*, and *N*. It is assumed that *A* and *P* have the same label and *A* and *N* have different labels.

In the training phase, for every Triplet in the training set, feature vectors $F_{A},\;F_{P},\;F_{N}$ are computed and then passed through a sigmoid to obtain $Y_{A},\;Y_{P},\;Y_{N}$. At that point, the loss function is:

(2) $$L=max\left({\left|Y_{A}-Y_{P}\right|}_{2}-{\left|Y_{A}-Y_{N}\right|}_{2},-\xi \right),.$$

where ξ is a positive number, and ${\left|x\right|}_{2}$ is the Euclidean norm of the vector. In other words, the loss function encourages the network to create similar representations for samples in the same class and different representations for samples in different classes. ξ is the margin, the value used is 1 because in the fixed margin tests carried out it was the one that returned the best results.

In the testing phase, the process is exactly the same as described for the testing phase of cross-entropy loss.

### 2.3. Adam Variants

Introduced in [20,21], the widely used optimization method Adam (referred to as Base Adam in the experimental section) takes advantage of adaptive gradient and momentum to compute adaptive learning rates for each parameter. It makes use of the gradient at the current step, the exponential moving average of the gradient (first order moment), and the exponential moving average of the square of the gradient (second order moment).

Thus, the first moment $m_{t}$ and the second moment $u_{t}$ are defined as:

(3) $$m_{t}=\rho_{1}m_{t-1}+\left(1-\rho_{1}\right)g_{t}$$

(4) $$u_{t}=\rho_{2}u_{t-1}+\left(1-\rho_{2}\right)g_{t}^{2}$$

where the hyperparameters $\rho_{1}$ and $\rho_{2}$ represent the exponential decay rate for the first and second moment (set respectively to 0.9 and 0.99), $g_{t}$ is the gradient at time t, and the square on $g_{t}$ is meant to be calculated component-wise. The moments are initialized as $m_{0}=u_{0}=0$.

To avoid small values of the moving averages due to being initialized to zero, Adam includes a bias-corrected version of the first and second order moments:

(5) $${\hat{m}}_{t}=\frac{m_{t}}{\left(1-\rho_{1}^{t}\right)}$$

(6) $${\hat{u}}_{t}=\frac{u_{t}}{\left(1-\rho_{2}^{t}\right)}$$

The parameter update is computed as follow:

(7) $$\theta_{t}=\theta_{t-1}-\lambda \frac{{\hat{m}}_{t}}{\sqrt{{\hat{u}}_{t}}+\epsilon },$$

where λ is the learning rate and ϵ is a very small positive number used to avoid any division by zero (usually set to 10^−8^). The operations are supposed to be component-wise.

As noted in [22], Adam performs reasonably well in practice compared to other adaptive learning methods; however, Adam does not utilize the change in immediate past gradient information, a utilization that is incorporated in [22,23].

#### 2.3.1. DGrad

This variant, proposed in [23], makes use of the absolute difference between the current gradient $g_{t}$ and the moving average of the element-wise squares of the gradients:

(8) $$\Delta ag_{t}=\left|g_{t}-avg_{t}\right|$$

where $avg_{t}$ is the moving average of the component-wise squares of the gradient.

The absolute difference $\Delta ag_{t}$ is then normalized by its maximum component as follows:

(9) $$\Delta {\hat{ag}}_{t}=\frac{\Delta ag_{t}}{\max\left(\Delta ag_{t}\right)}$$

Then, $\xi_{t}$ is defined as:

(10) $$\xi_{t}=Sig\left(4\cdot \Delta {\hat{ag}}_{t}\right)$$

where Sig(Δ) is the sigmoid function:

(11) $$Sig\left(x\right)=\frac{1}{1+e^{-x}}$$

Each parameter of the network is finally updated following the equation:

(12) $$\theta_{t+1}=\theta_{t}-\lambda \cdot \xi_{t}\frac{{\hat{m}}_{t}}{\sqrt{{\hat{u}}_{t}}+\epsilon }$$

where ${\hat{m}}_{t}$ and ${\hat{u}}_{t}$ are the first and second order moments seen in Adam.

#### 2.3.2. DecayDGrad (New)

This DGrad variant introduces a learning rate decay, both locally and in the whole training process. The local decay can be achieved with a periodic impulse, defined as follows:

(13) $$imp_{t}=e^{-{\left(\frac{2\times mod\left(t,s\right)}{s}\right)}^{2}}$$

where s=10 is the period (number of iterations between each impulse).

The impulse $imp_{t}$ is then multiplied by a global decay factor $d_{t}$, shown in the equation:

(14) $$d_{t}=e^{-\frac{2\times {\left(t-c\cdot n_{iter}\right)}^{2}}{n_{iter}^{2}}}$$

where $n_{iter}$ is the total number of iterations in the training process. The parameter c=0.25, multiplied by $n_{iter}$, determines the iteration whereby $d_{t}$ assumes its maximum value.

The parameter $\xi_{t}$ is therefore defined as:

(15) $$\xi_{t}=Sig\left(4\cdot \Delta \hat{ag_{t}}\right)\cdot imp_{t}\cdot d_{t}.$$

Each parameter of the network is updated as shown in (12).

Notice that $imp_{t}$ only has values in range 0 to 1, and its maximum value is assumed for iterations, which are multiples of s. The purpose of these restraints is to attenuate the value calculated by DGrad locally, namely progressively in the span of 𝑠 iterations, to get a better evaluation of the local minimum, thereby avoiding an eventual overshoot of the global minimum.

**Figure 4:**
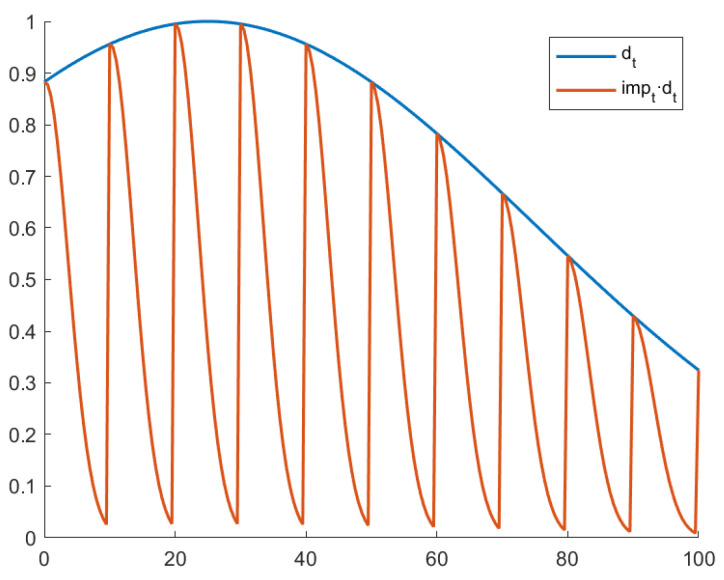
Plot of $d_{t}$ and $imp_{t}\cdot d_{t}$.

The reason behind the learning rate decay factor $d_{t}$ is to keep the learning rate high in the initial part of the training, which accelerates training and avoids the memorization of noisy data while at the same time extending the decay in later iterations. In this way, DGrad can learn complex patterns, as shown in [24]. The plot of $d_{t}$ and $imp_{t}\cdot d_{t}$ is reported in Figure 4.

## 3. Data Sets

The following five image data sets, representing very different classification tasks, were selected to demonstrate the versatility of the proposed method:

- BIRDz [25]: This balanced data set is a real-world benchmark for bird species vocalizations. The testing protocol is ten runs using the data split in [25]. The audio tracks were extracted from the Xeno-Canto Archive (http://www.xeno-canto.org/ (accessed on 25 August 2021)). BIRDz contains a total of 2762 acoustic samples from eleven North American bird species, along with 339 unclassified audio samples (consisting of noise and unknown bird vocalizations). The bird classes vary in size from 246 to 259. Each observation is represented by five spectrograms: (1) constant frequency, (2) frequency modulated whistles, (3) broadband pulses, (4) broadband with varying frequency components, and (5) strong harmonics.
- CAT [26,27]: This data set has ten balanced classes of cat vocalizations, with each one containing ~300 samples for a total of 2962 samples taken from Kaggle, Youtube, and Flickr. The testing protocol is 10-fold cross-validation. The average duration of each sample is 4 s.
- InfLar [28]: This data set contains eighteen Narrow-Band Imaging (NBI) endoscopic videos of eighteen different patients with laryngeal cancer. The videos were retrospectively analyzed and categorized into four classes (informative, blurred, containing saliva or specular reflections, and underexposed). The average video length is 39 s. The videos were acquired with an NBI endoscopic system (Olympus Visera Elite S190 video processor and an ENF-VH rhino-laryngo videoscope) with a frame rate of 25 fps and an image size of 1920 × 1072 pixels. A total of 720 video frames, 180 for each of the four classes, were extracted and labeled. The testing protocol is three-fold cross-validation with data separated at the patient level to ensure that the frames from the same class were classified based on the features characteristic of each class and not due to features linked to the individual patient (e.g., vocal fold anatomy).
- RPE [29]: This is a medical image classification data set that intends to distinguish the maturation of human stem cell-derived retinal pigmented epithelium. RPE is based on 195 images that were divided into sixteen subwindows. These subwindows were then assigned to one of four classes: (1) Fusifors, (2) Epithelioid, (3) Cobblestone, and (4) Mixed. Subwindows that were out of focus or that contained background information exclusively were discarded. This division of images into four and the exclusion process produced a total of 1862 images.
- Port [30]: This data set contains 927 paintings from six different art movements: (1) High Renaissance, (2) Impressionism, (3) Northern Renaissance, (4) Post-Impressionism, (5) Rococo, and (6) Ukiyo-e. Ten-fold cross-validation is the testing protocol.

The same testing protocol presented in the papers introducing each data set is used in the experimental section, with accuracy being the performance indicator.

## 4. Experimental Results

The default settings in the MATLAB framework for Siamese networks were used to train the SNNs in all experiments to ensure no overfitting for any given data set. For Adam optimization and its variants, the number of iterations was set to 3000 with no stop criterion, the gradient decay factor to 0.9, the squared gradient decay factor to 0.99, and the learning rate to 0.0001.

The first run of experiments is reported in Table 1. In these tests, we used all the data sets. Each performance cell in Table 1 contains three rows of values for each data set:

1. Top: The performance obtained using the method named FULLY for SVM input;
2. Middle: The performance obtained using the method named DEEPER for SVM input;
3. Bottom: The fusion by average rule of the SVMs in 1 and 2.

The last row in Table 1 reports average performance of each approach of that column.

The clustering method is k-means for all methods, and the number of prototypes is in the set (15, 30, 45, 60). Thus, four networks are trained using the four numbers of prototypes in the set; the four SVMs trained in this way are combined by average rule.

For the sake of computation time, we used a single network topology in this test, which is the first topology tested in [14] and the Siamese topology recommended by Mathworks (see the Appendix A).

- The columns of Table 1 report the following approaches:
- Cross: Binary Cross Entropy loss function coupled with base Adam (this is the best approach proposed [14]);
- CrossDD: Binary Cross Entropy loss function coupled with our new Adam variant DecayDGrad;
- Triplet: Triplet loss function coupled with base Adam.
- X + Y (columns 5 and 6): the fusion between X and Y.

From the results reported in Table 1, the following conclusions can be drawn:

- Triplet produces a result that is similar to Cross on three data sets but performs better than Cross in InfLar and worst in CAT;
- The fusion between Cross and Triplet boosts the performance of the base loss functions, except in the case of CAT;
- The fusion among all the different approaches (see bottom cells in the column Triplet+Cross and Triplet+Cross+CrossDD) produces the best average performance.

Table 2 reports results using combinations of the two loss functions on all eight topologies. Because running experiments on all five data sets was computationally too expensive, we chose to run them only on InfLar and Port because they are very different application problems.

In each cell of Table 2, the following four results are reported:

1. Top: Cross function coupled with FULLY for SVM input (the best approach proposed in [14]);
2. Upper: Triplet loss function coupled with FULLY for SVM input;
3. Lower: Fusion by average rule among Cross coupled with FULLY, Cross coupled with DEEPER, Triplet coupled with FULLY, and Triplet coupled with DEEPER;
4. Bottom: This is the fusion by average rule of SVMs 1 and 2 described for the method reported at the bottom of Table 1 but with the addition of CrossDD coupled with both FULLY and DEEPER.

The last row of Table 2 reports the fusions of #4 above for the numbered topologies.

In [13], we showed that combining more than four networks using the same topology (but varying the clustering algorithm) failed to improve performance. Examining Table 2, we discovered that changing the loss function and the method for building the dissimilarity space is beneficial when making an ensemble. We also observed that for all topologies except #6 in the Portrait data set (Port), the best performance is not obtained by contrastive loss coupled with FULLY (as was the case in [14]); instead, on average, the new method DEEPER succeeds in boosting performance. Finally, we learned that adding CrossDD, our new Adam variant, to the ensemble for InfLar generally does not increase performance; CrossDD works very well with the first topology but performs worst with the other topologies. On Port, however, the addition of CrossDD generally does improve performance.

In Table 3, we compare our best results on InfLar and Port with the best ensembles reported in [12,13,14] that tested ensembles of SNNs and CNN subnets using all eight topologies. In addition, the performance of four well-known CNNs is reported for baseline comparison, along with their fusion (eCNN) by average rule. The fine-tuning of the CNNs pretrained on ImageNet was performed with the following training options: batch size: 30; max epoch: 20 (for all the networks with no freezing). The row “Fusion x-y + eCNN” is the sum rule between Fusion x-y (see Table 2) and eCNN. Before the fusion, the score of Fusion x-y and eCNN are normalized to mean 0 and standard deviation 1.

As can be observed in Table 3, the proposed ensembles outperform previous methods based on Siamese networks and boosts the performance of the ensemble of CNNs. On the data set InfLar, the performance of the best standalone topology (see Table 2) is 92.78, which is comparable with the performance obtained by a CNN; however, on the Port data set, where our new Adam variant increased performance, the performance gap between the CNNs and Siamese networks is still significant. The approach proposed in this work also greatly improves previous Siamese methods applied to this data set.

Finally, in Table 4 we report the training time (seconds) of Siamese networks, in the InfLar data sets, considering the different topologies. The training time is computed using a GTX1080. Both the loss functions here used are considered in Table 4.

## 5. Conclusions

This paper proposes an image classification system that, like several recent studies, generates dissimilarity spaces from which features are extracted and trained on a set of SVMs. The objective of this study was to produce a high performing ensemble of Siamese networks based on combining different topologies, loss functions, and optimization methods (with one new Adam variant proposed here) from which features could be extracted for training the SVMs.

Results on five cross-domain image data sets demonstrate the superior power of the proposed approach compared with previous works using ensembles of Siamese networks. Comparison with the state-of-the-art confirms that the fusion of the different topologies, loss functions, and optimization approach methods is a feasible way for generating a robust and highly generalizable image classification system.

In the future, we intend to validate our approach on additional cross-domain image data sets and investigate more techniques for building an ensemble of Siamese networks.

## Figures and Tables

**Table 1:** Performance of the two tested loss functions (boldface represents the best performance).

	Cross	CrossDD	Triplet	Triplet + Cross	Triplet + Cross + CrossDD
CAT	**83.05**	80	77.29	82.37	**83.05**
	69.15	71.19	72.2	71.53	76.27
	80	79.32	76.61	80	80
InfLar	86.94	93.75	90.42	91.25	**93.47**
	87.78	88.33	88.89	89.17	91.39
	89.44	92.36	90.56	91.39	92.64
BIRDz	94.49	93.35	94.08	94.9	94.56
	92.92	92.53	94.02	94.36	94.16
	94.52	93.91	94.84	**95.21**	94.88
RPE	84.52	84.58	85.43	85.75	84.97
	84.15	84.49	85.1	85	85.16
	84.73	85.08	85.17	**85.92**	85.48
Port	70.99	74.44	68.72	72.82	74.33
	69.57	70.54	70.96	71.83	73.22
	70.55	74.53	72.59	74.11	**74.65**
Average	82.85	83.89	83.12	84.37	**85.21**

**Table 2:** Performance varying the network topologies (topologies are described in [14] and reprinted in the Appendix A; boldface represents the best performance).

Topology	InfLar	Port
**Topology 1**	86.94	70.99
	90.42	68.72
	91.39	74.11
	**92.64**	**74.65**
**Topology 2**	85.56	68.73
	**92.78**	70.02
	92.08	72.49
	91.67	72.92
**Topology 3**	79.44	60.23
	83.75	68.17
	**85.42**	68.41
	84.03	**69.17**
**Topology 4**	87.50	69.69
	91.25	68.29
	**92.22**	73.58
	90.97	74.65
**Topology 5**	84.03	60.00
	**89.44**	65.03
	87.64	64.95
	85.14	**69.69**
**Topology 6**	87.64	**73.48**
	88.61	68.07
	**91.25**	73.25
	90.56	73.24
**Topology 7**	79.44	66.03
	**91.39**	70.85
	85.00	70.85
	84.72	71.85
**Topology 8**	86.39	65.58
	87.22	66.55
	**91.11**	66.55
	90.56	72.16
**Fusion 1–4**	**92.78**	**75.09**
**Fusion 1–6**	91.53	74.45
**Fusion 1–8**	91.81	74.98

**Table 3:** Performance accuracy obtained considering different standard CNNs and other Siamese approaches (xxx * means that it does not converge, and boldface represents best performance).

Method	InfLar	Port
[12]	74.86	xxx *
[13]	89.86	71.42
[14]	91.10	73.05
Fusion 1–4	92.78	75.09
Fusion 1–6	91.53	74.45
Fusion 1–8	91.81	74.98
GoogleNet	90.42	80.38
VGG16	91.53	86.51
VGG19	92.22	82.42
GoogleNetP365	93.61	80.91
eCNN	94.03	86.41
Fusion 1–4 + eCNN	**94.44**	**86.84**
Fusion 1–6 + eCNN	**94.44**	**86.84**
Fusion 1–8 + eCNN	94.31	**86.84**

**Table 4:** Computation time for training a single Siamese network, each column reports the computation time of a given topology network, numbered 1–8 (topologies are described in [14] and reprinted in the Appendix A).

InfLar	1	2	3	4	5	6	7	8
Cross	1029	2009	317	1179	512	580	679	559
Triplet	1500	2721	400	1620	678	752	885	725

## Data Availability

All data sets are publicly available and the source code is located at https://github.com/LorisNanni/Closing-the-performance-gap-between-siamese-networks-for-dissimilarity-image-classification-and-conv (accessed on 24 August 2021).

## Appendix A

For ease of reference, a description of the eight SN networks in [14] are reprinted below. CNN Siamese Networks (1–8) layers. The bold layers are those used for feeding DCT.

**Siamese Network 1**				
**Layers**	**Activations**	**Learnable**	**Filter Size**	**Num. of Filters**
Input Layer	224 × 224			
2D Convolution	215 × 215 × 64	6464	10 × 10	64
ReLU	215 × 215 × 64	0		
Max Pooling	107 × 107 × 64	0	2 × 2	
2D Convolution	101 × 101 × 128	401,536	7 × 7	128
ReLU	101 × 101 × 128	0		
Max Pooling	50 × 50 × 128	0	2 × 2	
2D Convolution	47 × 47 × 128	262,272	4 × 4	128
ReLU	47 × 47 × 128	0		
Max Pooling	23 × 23 × 128	0	2 *×* 2	
2D Convolution	19 × 19 × 64	204,864	5 *×* 5	64
ReLU	19 × 19 × 64	0		
Fully Connected	4096	94,638,080		
**Siamese Network 2**				
**Layers**	**Activations**	**Learnable**	**Filter Size**	**Num. of Filters**
Input Layer	224 × 224	0		
2D Convolution	220 × 220 × 64	1664	5 *×* 5	64
LeakyReLU	220 × 220 × 64	0		
2D Convolution	216 × 216 × 64	102,464	5 *×* 5	64
LeakyReLU	216 × 216 × 64	0		
Max Pooling	108 × 108 × 64	0	2 *×* 2	
2D Convolution	106 × 106 × 128	73,856	3 *×* 3	128
LeakyReLU	106 × 106 × 128	0		
2D Convolution	104 × 104 × 128	147,584	3 *×* 3	128
LeakyReLU	104 × 104 × 128	0		
Max Pooling	52 × 52 × 128	0	2 *×* 2	
2D Convolution	49 × 49 × 128	262,272	4 *×* 4	128
LeakyReLU	49 × 49 × 128	0		
Max Pooling	24 × 24 × 128	0	2 *×* 2	
2D Convolution	20 × 20 × 64	204,864	5 *×* 5	64
LeakyReLU	20 × 20 × 64	0	5 *×* 5	
Fully Connected	2048	52,430,848		
**Siamese Network 3**				
**Layers**	**Activations**	**Learnable**	**Filter Size**	**Num. Filters**
Input Layer	224 × 224			
2D Convolution	55 × 55 × 128	6400	7 *×* 7	128
Max Pooling	27 × 27 × 128	0	2 *×* 2	
2D Convolution	23 × 23 × 256	819,456	5 *×* 5	256
ReLU	23 × 23 × 256	0		
2D Convolution	19 × 19 × 128	819,328	5 *×* 5	128
Max Pooling	9 × 9 × 128	0	2 *×* 2	
2D Convolution	7 × 7 × 64	73,792	3 *×* 3	64
ReLU	7 × 7 × 64	0		
Max Pooling	3 × 3 × 64	0	2 *×* 2	
Fully Connected	4096	2,363,392		
**Siamese Network 4**				
**Layers**	**Activations**	**Learnable**	**Filter Size**	**Num. of Filters**
Input Layer	224 × 224			
2D Convolution	218 × 218 × 128	6400	7 *×* 7	128
Max Pooling	54 × 54 × 128	0	4 *×* 4	
ReLU	54 × 54 × 128	0		
2D Convolution	50 × 50 × 256	819,456	5 *×* 5	256
ReLU	50 × 50 × 256	0		
2D Convolution	48 × 48 × 64	147,520	3 *×* 3	64
Max Pooling	24 × 24 × 64	0	2 *×* 2	
2D Convolution	22 × 22 × 128	73,856	3 *×* 3	128
ReLU	22 × 22 × 128	0		
2D Convolution	18 × 18 × 64	204,864	5 *×* 5	64
Fully Connected	4096	84,938,752		
**Siamese Network 5**				
**Layers**	**Activations**	**Learnable**	**Filter Size**	**Num. of Filters**
Input Layer	224 × 224			
2D Convolution	215 × 215 × 64	6464	10 *×* 10	64
Max Pooling	107 × 107 × 64	0	2 *×* 2	
ReLU	107 × 107 × 64	0		
2D Convolution	26 × 26 × 128	401,536	7 *×* 7	128
ReLU	26 × 26 × 128	0		
2D Convolution	9 × 9 × 128	409,728	5 *×* 5	128
ReLU	9 × 9 × 128	0		
2D Convolution	6 × 6 × 64	131,136	4 *×* 4	64
ReLU	6 × 6 × 64	0		
Fully Connected	4096	9,441,280		
**Siamese Network 6**				
**Layers**	**Activations**	**Learnable**	**Filter Size**	**Num. of Filters**
Input Layer	224 × 224			
2D Convolution	218 × 218 × 64	3200	7 *×* 7	64
Max Pooling	109 × 109 × 64	0	2 *×* 2	
ReLU	109 × 109 × 64	0		
2D Convolution	107 × 107 × 128	73,856	3 *×* 3	128
Max Pooling	53 × 53 × 128	0	2 *×* 2	
ReLU	53 × 53 × 128	0		
2D Convolution	53 × 53 × 64	8256	1 *×* 1	64
ReLU	53 × 53 × 64	0		
2D Convolution	51 × 51 × 128	73,856	3 *×* 3	128
ReLU	51 × 51 × 128	0		
Max Pooling	25 × 25 × 128	0	2 *×* 2	
2D Convolution	25 × 25 × 128	16,512	1 *×* 1	128
ReLU	25 × 25 × 128	0		
2D Convolution	22 × 22 × 64	131,136	4 *×* 4	64
Max Pooling	11 × 11 × 64	0	2 *×* 2	
ReLU	11 × 11 × 64	0		
Fully Connected	4096	31,723,520		
**Siamese Network 7**				
**Layers**	**Activations**	**Learnable**	**Filter Size**	**Num. of Filters**
Input Layer	224 × 224			
Dropout Layer	224 × 224	0		
2D Convolution	218 × 218 × 64	3200	7 *×* 7	64
Max Pooling	109 × 109 × 64	0	2 *×* 2	
2D Convolution	105 × 105 × 128	204,928	5 *×* 5	128
Max Pooling	52 × 52 × 128	0	2 *×* 2	
2D Convolution	48 × 48 × 64	204,864	5 *×* 5	64
Max Pooling	24 × 24 × 64	0	2 *×* 2	
2D Convolution	22 × 22 × 256	147,712	3 *×* 3	256
Max Pooling	11 × 11 × 256	0	2 *×* 2	
Fully Connected	4096	16,781,312		
**Siamese Network 8**				
**Layers**	**Activations**	**Learnable**	**Filter Size**	**Num. of Filters**
Input Layer	224 × 224			
2D Convolution	215 × 215 × 32	3232	10 *×* 10	32
Max Pooling	107 × 107 × 32	0	2 *×* 2	
ReLU	107 × 107 × 32	0		
2D Grouped Convolution	101 × 101 × 64	50,240	7 *×* 7	64
2D Convolution	97 × 97 × 128	204,928	5 *×* 5	128
Max Pooling	48 × 48 × 128	0	2 *×* 2	
ReLU	48 × 48 × 128	0		
2D Grouped Convolution	46 × 46 × 256	147,712	3 *×* 3	256
Fully Connected	4096	2,218,790,912		
